# 2440: Among heart failure patients, cannabis use is an independent predictor of hospitalization and discharge against medical advice: Data from the 2012 Nationwide Emergency Department Sample (NEDS)

**DOI:** 10.1017/cts.2017.108

**Published:** 2018-05-10

**Authors:** Adeyinka C. Adejumo, Samson Alliu, Nnaemeka Onyeakusi, Tokunbo Opeyemi Ajayi, Adegbala Oluwole Muyiwa, Akintunde Akinjero, Kelechi Adejumo, Edgar Lichstein

## Abstract

OBJECTIVES/SPECIFIC AIMS: To assess the effect of cannabis on impaired judgment and health outcomes among heart failure patients in the emergency room. METHODS/STUDY POPULATION: Patients with heart failure presenting to the emergency room. Cannabis with confounders such as income level, insurance type, tobacco use, and age. Discharged against medical advice to assess impaired judgment. Hospitalization rates, length of stay, and death rate to assess health outcomes. Multivariate logistic regression to access the odds of each of these outcomes from cannabis RESULTS/ANTICIPATED RESULTS: Cannabis is associated with impaired outcome (increase in discharge against medical advice). Cannabis have poorer health outcome in terms of more hospitalizations from the emergency department. Cannabis also have better health outcome in terms of shorter length of stay and death rate among cannabis users Versus nonusers. DISCUSSION/SIGNIFICANCE OF IMPACT: This is crucial to inform health care providers to ensure better counseling of cannabis users. This result should also be considered to interpret other publications that shows better outcomes in patients taking cannabis. Cannabis users might only seem to have a better outcome because they tend to discharge against medical advice and thereby die outside the hospital, etc.

